# Sugar, fructose, uric acid and hypertension in children and adolescents

**DOI:** 10.1186/1824-7288-41-S2-A76

**Published:** 2015-09-30

**Authors:** Francesca Viazzi, Simonetta Genovesi, Maria Amalia Ambruzzi, Marco Giussani

**Affiliations:** 1 University of Genoa, Department of Internal Medicine, IRCCS, AUO San Martino-IST, Genova, Italy; 2 Department of Health Sciences, University of Milano-Bicocca and Nephrology Unit, San Gerardo Hospital, Monza, Italy; 3 Ospedale Pediatrico IRCCS Bambino Gesù, Rome, Italy; 4 Family Pediatrician, Milan, Italy

## 

Fructose consumption has been increasing over recent decades and is believed to play a role in the rising epidemic of metabolic disorders and hypertension (HT) in children [1,2]. This theory is supported by epidemiological and experimental studies in animals and humans.

High-fructose diets upregulate sodium and chloride transporters, resulting in salt overload that increases blood pressure (BP) [3]. Moreover, excess fructose has also been found to deregulate vasoconstrictors and vasodilators, and over-stimulate the sympathetic nervous system. Metabolism of fructose is mediated by fructokinase, which uses ATP as a phosphate donor. Unlike glucose, there is no feedback mechanism regulating fructokinase. As a result, AMP is continuously involved in the production of uric acid (UA) [4]. In adolescents in the US, serum UA was showed to increase from the lowest to the highest category of fructose-sweetened beverage intake and this increment was paralleled by an increase in BP, even independently of body mass index [5]. These data suggest pathways other than obesity relating soft drinks to the development of HT.

Epidemiological studies demonstrate an association between serum UA and both prevalence and new onset of essential HT in adolescents [6]. Recently, UA showed a strong independent relationship with BP values across different BP categories, from normal BP up to pre-and finally to established HT in children at relatively high cardiovascular risk [7].

Animal models support a two-phase mechanism for the development of hyperuricemic HT. Initially, UA induces vasoconstriction by activation of the renin-angiotensin system and reduction of nitric oxide. Over time, UA uptake into vascular smooth muscle cells (VSMC) causes cellular proliferation and arteriolosclerosis that impair pressure natriuresis, causing sodium-sensitive HT [8]. Increased UA causes endothelial dysfunction by inflicting oxidative stress once inside cells. UA internalization is mediated by URAT-1, and stimulates production of growth factors and chemokines in human VSMC [9] and increases ROS activating NADPH-oxidase, leading to apoptosis in human tubular cells [10]. These actions may, at least in part, explain the association between increased serum UA levels and initial cardiovascular and renal damage described in adolescents with obesity or HT [11,12].

Interestingly, in both animal and human studies, allopurinol attenuates the development of fructose-induced HT by lowering UA. Indeed, lowering UA with either allopurinol or probenecid reduces BP in adolescents with HT or pre-HT [13,14]. While larger studies are needed, fructose assumption and serum UA are emerging as potentially modifiable risk factors for the prevention and treatment of HT in children.

## References

[B1] Choi JW Ford ES Gao X Choi HK Sugar-sweetened soft drinks, diet soft drinks, and serum uric acid level: the Third National Health and Nutrition Examination Survey Arthritis Rheum 2008 59 1 109 116 10.1002/art.23245 18163396

[B2] Grimes CA Riddell LJ Campbell KJ Nowson CA Dietary salt intake, sugar-sweetened beverage consumption, and obesity risk Pediatrics 2013 131 1 14 21 10.1542/peds.2012-1628 23230077

[B3] Singh AK Amlal H Haas PJ Dringenberg U Fussell S Barone SL Fructose-induced hypertension: essential role of chloride and fructose absorbing transporters PAT1 and GLUT5 Kidney Int 2008 74 4 438 447 10.1038/ki.2008.184 18496516 PMC10947803

[B4] Stirpe F Della Corte E Bonetti E Abbondanza A Abbati A De Stefano F Fructose-induced hyperuricaemia Lancet 1970 2 7686 1310 1311 4098798 10.1016/s0140-6736(70)92269-5

[B5] Nguyen S Choi HK Lustig RH Hsu CY Sugar-sweetened beverages, serum uric acid, and blood pressure in adolescents J Pediatr 2009 154 6 807 813 10.1016/j.jpeds.2009.01.015 19375714 PMC2727470

[B6] Feig DI Uric acid and hypertension Semin Nephrol 2011 31 5 441 446 10.1016/j.semnephrol.2011.08.008 22000651

[B7] Viazzi F Antolini L Giussani M Brambilla P Galbiati S Mastriani S Serum uric acid and blood pressure in children at cardiovascular risk Pediatrics 2013 132 1 e93 e99 10.1542/peds.2013-0047 23776119

[B8] Feig DI Madero M Jalal DI Sanchez-Lozada LG Johnson RJ Uric acid and the origins of hypertension J Pediatr 2013 162 5 896 902 10.1016/j.jpeds.2012.12.078 23403249 PMC7556347

[B9] Kang DH Han L Ouyang X Kahn AM Kanellis J Li P Uric acid causes vascular smooth muscle cell proliferation by entering cells via a functional urate transporter Am J Nephrol 2005 25 5 425 433 10.1159/000087713 16113518

[B10] Verzola D Ratto E Villaggio B Parodi EL Pontremoli R Garibotto G Uric acid promotes apoptosis in human proximal tubule cells by oxidative stress and the activation of NADPH oxidase NOX 4 PLoS One 2014 9 12 e115210 10.1371/journal.pone.0115210 25514209 PMC4267812

[B11] Pacifico L Cantisani V Anania C Bonaiuto E Martino F Pascone R Serum uric acid and its association with metabolic syndrome and carotid atherosclerosis in obese children Eur J Endocrinol 2009 160 1 45 52 10.1530/EJE-08-0618 18952765

[B12] Tomczak J Wasilewska A Milewski R Urine NGAL and KIM-1 in children and adolescents with hyperuricemia Pediatr Nephrol 2013 28 9 1863 1869 10.1007/s00467-013-2491-y 23673972 PMC3722436

[B13] Feig DI Soletsky B Johnson RJ Effect of allopurinol on blood pressure of adolescents with newly diagnosed essential hypertension: a randomized trial JAMA 2008 300 8 924 932 10.1001/jama.300.8.924 18728266 PMC2684336

[B14] Soletsky B Feig DI Uric acid reduction rectifies prehypertension in obese adolescents Hypertension 2012 60 5 1148 1156 10.1161/HYPERTENSIONAHA.112.196980 23006736

